# Small-scale irrigators’ intentions to adapt water use behaviour to climate variability in South Africa

**DOI:** 10.1371/journal.pone.0351125

**Published:** 2026-07-23

**Authors:** Rachel M. Msila, Yonas T. Bahta, Henry Jordaan, Markus A. Monteiro

**Affiliations:** Department of Agricultural Economics, University of the Free State, Bloemfontein, South Africa; Universidade Federal de Mato Grosso do Sul, BRAZIL

## Abstract

This study aimed to determine the impacts of climate variability on smallholder maize producers’ water use behaviour and the factors that influence their decision-making in the Vhembe District Municipality, Limpopo Province, South Africa. This study utilized a survey from 366 smallholder maize irrigators, Theory of Planned Behaviour (TPB), and Structural Equation Modelling (SEM). The TPB posits that behavioural intentions can influence actual behaviour of individuals and groups, and it consists of three behavioural factors, namely attitude, subjective norms, and perceived behavioural control. Findings indicated that most of the farmers (96%) in the study area were dependent on farming as their primary occupation and livelihood. Most of the farmers were senior citizens with the mean age being between 51–60 and above 61 years of age, majority of the respondents (71%) had secondary education level and majority of the farmers (40%) had less than 10 years of farming experience and 35% had between 11–20 years of farming experience. In addition, the findings revealed that attitude, subjective norms, and perceived behavioural control significantly influence the small-scale farmers’ intentions to adapt to climate variability. The results suggest that as farmers’ attitudes become positive, their intentions to adapt enhance, and the perceptions of adaptation behaviours have a significant impact on individual farmers’ willingness to adapt. In addition, attitude and subjective norms have a substantial influence on the intentions of behavioural changes of farmers’ adaptation. However, perceived behavioural control does influence Intention but has a minor impact compared to attitudes and norms. Therefore, socio-psychological issues should be taken into consideration to improve smallholder farmers’ adoption of sustainable water use practices. This could raise smallholders’ livelihoods and agricultural productivity.

## 1. Introduction

Water scarcity is severe in semi-arid areas, which makes it difficult to secure water supplies due to high populations and industrial activities [1]. In light of these challenges, climate variability further exacerbates competition for water among key sectors such as agriculture, industry, and domestic use. As a result, effective water management strategies and policy interventions have become increasingly essential to ensure equitable distribution and sustainable utilization of water resources [2].

Water use behaviour refers to the behavioural Intention of an individual to conserve water daily [3]. Water use behaviour refers to the behavioural Intention of an individual to conserve water in daily activities [3]. Monteiro et al. [4] conducted a systematic review of water use in agricultural contexts and identified six key behavioural domains: (1) adaptation to climate variabiliy; (2) adoption of water-saving technologies; (3) implementation of water conservation techniques or practices; (4) compliance with water-saving policies; (5) water-use behaviour in relation to institutional performance; and (6) seasonal irrigation water usage patterns. Among these, adaptation to climate variability has emerged as a critical behavioural response, particularly for smallholder farmers who are highly vulnerable to climatic variability. Understanding how farmers’ water uses behaviours, especially adaptive behaviours, are influenced by climate variability is essential for ensuring adequate agricultural output and sustainable resource utilization [3]. The decision-making process of farmers on how to better utilize the available water resources is vital to determine the factors that contribute to their water-use behaviour to mitigate the effects of climate variability [5].

South Africa is one of the top 30 driest nations on earth, with 450 mm of yearly precipitation. The nation experiences the least amount of rainfall compared to the worldwide average of 860 mm [6]. South Africa’s semi-arid environment and the regional variations in rainfall have made water demand a critical concern. Consumers in South Africa primarily get their water from rainfall and the few clean streams that are available. Thus, the nation can be regarded as one with limited access to water resources [7], given the fact that water can no longer be adequately considered a renewable natural resource, given the existing patterns of water demand. As a result of increasing agricultural production to sustain the growing population and the impacts of climate variability, fresh water, which only makes up 2.5% of the planet’s water, is in danger. Therefore, to lessen this threat, water conservation techniques and effective management are becoming more and more critical, with farmers’ behaviour being a primary focus [5].

Understanding how actors in the agricultural sector use water is essential for evaluating the efficiency and sustainability of irrigation practices [4,5]. Despite the central role of farmers in water management, their behavioural responses and decision making under conditions of water scarcity remain insufficiently understood, particularly across diverse agricultural contexts and among smallholder farming systems [5]. The sustainability and availability of water may be impacted by farmers’ behaviours when using water; therefore, water conservation strategies are becoming essential to ensure farmers use water wisely. Determining the factors influencing farmers’ decisions on the use of water by each stakeholder in the agricultural sector and how those decisions impact the availability of water resources is a challenging task. According to Bwapwa [8], the irrigation industry in South Africa uses the most water (62%), but it also contributes to between 30% and 40% of water loss. The desire to create technologies that could increase the water efficiency of irrigated agriculture is driven by the ongoing demand for water supplies. The identified technical advancements have the potential to alleviate the pressure of water shortages, especially in regions where there is intense competition between urban and agricultural consumers [9].

In the Southern African region and South Africa, the most utilised crop for human consumption is white maize, at 60% production output, and yellow maize is produced at 40% for animal feed production [10]. White maize has many uses in the form of grain, meal, and green mielies for sale in the form of fresh cobs on the market. Maize is considered the main staple food and the most extensively grown crop in South Africa, followed by soya bean, wheat, sunflower, grain sorghum, and other legume crops [11]. Maize is produced mainly under dryland conditions, with 10% made under irrigation. However, it can be regarded as an essential grain crop under irrigation as it produces high yields [12]. Therefore, it can be considered as one of the most efficient grain crops in terms of water usage, within a relatively short period of 100–120 days [7]. Consequently, maize production by smallholder farmers has been declining due to harsh climate variations and the lack of climate-smart technology. Capital constraints and lack of government support play a huge role in the lack of innovative adoption methods to mitigate the impacts of climate variability on their farms [13].

Droughts and water restrictions have not changed the patterns of water use consumption [14]. By positively changing the farmers’ decision-making process to use water more efficiently and effectively, farmers can expand crop yields, reduce water misuse, and mitigate environmental damage [15]. To enhance sustainable water use, especially changing water use behaviour, the decision-making process of smallholder farmers would have to adjust to understanding and commitment of all farmers to develop a culture of water conservation [16]. The main objectives of this study were to determine the response to climate variability on smallholder maize irrigators’ intentions to adapt water use behaviours by determining how attitudes, subjective norms, and perceived behavioural control influence the smallholder farmers’ decision-making to implement sustainable water use practices.

Section 2 will describe the materials and techniques used, such as the survey, the Theory of Planned Behaviour (TPB), and structural equation modelling (SEM). Furthermore, the results are presented in Section 3, and the findings are discussed in Section 4. Summing up section 5 provides crucial insights and recommendations as it concludes the study.

## 2. Materials and methods

### 2.1. Study area

With five district municipalities and 22 local municipalities, the Limpopo Province makes up almost 10% of South Africa’s total surface area. It is the main route connecting South Africa to nations outside Sub-Saharan Africa. The province’s capital is Polokwane, and 12% of its people live in cities, with the remaining 88% being in the province’s rural central and eastern regions [17]. The physical distribution of the traditional authority regions throughout the province is closely associated with the settlement pattern of the province of Limpopo. A total of 150 traditional authorities govern 25% of the province’s land. Although there is still difficulty with spatial integration, the greater concentration of settlements is still the most distant from the urban amenities and key corridors [18].

The area selected for the study was the Vhembe Local District Municipality, located in the Limpopo Province of South Africa. To the north lies Zimbabwe, to the northwest is Botswana, and to the southeast is Mozambique, all of which can be accessed through the Kruger National Park [19]. The district comprises four local municipalities: Thulamela Local Municipality, Makhado Local Municipality, Musina Local Municipality, and the recently established Collins Chabane Local Municipality. Within the district, there are four main towns: Makhado, Malamulele, Musina, and Thohoyandou [20].

**Fig 1:**
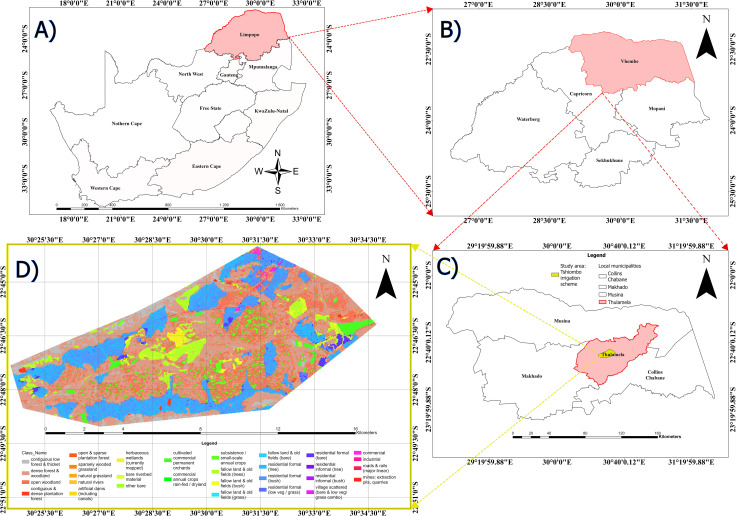
Spatial representation of the study area at the National, District, local, and site levels.

Fig 1 provides a multi-scale representation of the study area. Map “A” provides a national overview, showing the location of Limpopo province within South Africa, with Limpopo highlighted in red. Map “B” displays the district municipalities within Limpopo, with the Vhembe District Municipality (in Thohoyandou) highlighted in red to indicate the specific district where the study area is located. Map “C” provides an overview of the local municipalities within the Vhembe District Municipality, with the Thulamela Local Municipality highlighted in red, as this is where the study area occurs. Within the Thulamela (in Thohoyandou) Local Municipality in Map “C”, a yellow polygon represents the specific study site. Map “D” presents a zoomed-in view of this yellow polygon, providing a detailed land cover representation of the study area.

### 2.2. Research design

This study adopted a quantitative research design and approach to systematically examine the impact of climate variability on the water use behaviour of smallholder maize producers in the Vhembe District Municipality, Limpopo Province, South Africa. A deductive reasoning framework was used, guided by the TPB, to test hypotheses related to smallholder farmers’ decision-making and adaptation processes in response to climate variability. A cross-sectional survey was used to gather primary data, with structured Likert-scale questionnaires administered face-to-face to 366 smallholder maize irrigators.

Primary data was collected on a face-to-face basis from February to March 2024, with the questionnaires translated into Tshivenda to ensure accurate responses, which was the local language spoken by the farmers. The study examined behavioural determinants such as attitudes, subjective norms, and perceived behavioural control, which influence irrigation practices and adaptation responses. For data analysis, SEM was used, enabling rigorous hypothesis testing and evaluation of causal relationships between climate variability and smallholder farmers’ water use behaviour.

The study used Likert scale surveys, and the measurement items included in the questionnaires ranged from 1 to 5, with 1 = strongly disagree, 2 = disagree, 3 = neither, 4 = agree, and 5 = strongly agree. Each construct had separate climate variability and change measurement items. Prior literature and studies were consulted in the formulation of the questionnaire with reference to questions that were climate adaptation oriented. Prior to usage, pilot surveys were carried out to evaluate the reliability and consistency of the measurement items chosen. Following the collection of data from the pilot survey, the questionnaire underwent the required modifications and revisions before being finalized.

The purpose of the questions was to gauge farmers’ attitudes concerning changing and unpredictable climates on their farms. This study is part of a larger project entitled “Assessing the Social and Economic Impact of Changed Water-Use Behaviour in Selected Production and Irrigation Schemes in South Africa,” funded by the Water Research Commission (WRC) of South Africa (Project Number: C2022/2023 − 00798). Ethical clearance for the protocol was approved by the Research Ethics Committee at the University of the Free State in accordance with the General/Human Research Ethics (GHREC) guidelines and regulations. Reference number UFS-HSD2023/1327. Participants took part voluntarily after the ethical principles were publicly declared. All participants were informed, and they completed and signed a consent form before actively participating in the study.

To ensure the representativeness of the sample, according to the census of small-scale agriculture in Vhembe district (2023) [21], there were 1504 smallholder irrigated maize producers in the Vhembe District Municipality of Limpopo. From the 1504 farmers, 366 smallholder irrigated maize producers were chosen using the simple random sampling formula of Cochran [22] and Barlett et al. [23]. The correct sample size was determined using Cochran’s [22] sample size formula (Equation 1):


$$\begin{array}{c} \text{Sample size}=\frac{\left(\text{q}{)}^{\text{2}}*(\text{z})(\text{r}\right)}{(\text{w}{)}^{\text{2}}} \end{array}$$ (1)

Where, “q” is the level of confidence/alpha level (value for the selected alpha level indicates the level of risk the researcher is willing to take so that the actual margin of error may exceed the acceptable margin of error); (z) (r)-”z” and “r” are the estimates of the variance of the population; estimate of variance calculated as = 0.25 (maximum possible proportion (0.5)*1-maximum possible proportion (0.5) produces maximum possible sample size); and “w” is an acceptable margin of error for proportion being estimated = 0.05 (5%) (error researcher is willing to take).

If this formula is applied to the study with an alpha level of 1.65 (0.10), and the estimated variance of 0.5 and an error level of 0.05 are used, the formula would be as follows (Equation 2):


$$\begin{array}{c} \text{Sample size}=\frac{\left(\text{1}.\text{65}{)}^{\text{2}}*(\text{0}.\text{5})(\text{0}.\text{5}\right)}{(\text{0}.\text{05}{)}^{\text{2}}} \end{array}$$ (2)

Sample size = 272 (resulting in a sample size of 272 respondents)

As mentioned above, according to the census of small-scale agriculture in Vhembe district (2023) [21], there were 1504 smallholder irrigated maize producers in the Vhembe District Municipality of Limpopo. The application of this formula (Equations 3 and 4) revealed that a sample size of at least 231 respondents would ensure accurate results.


$$\begin{array}{c} \text{ N1 }=\frac{\text{Sample size}}{\text{1}\;+\;(\text{ N0}/\text{population})} \end{array}$$ (3)

$$\begin{array}{c} N\text{1}=\frac{\text{272}}{\text{1}\;+\;(\text{ 272}/\text{1504})} \end{array}$$ (4)

*N*1 = 231

The study obtained complete sets of questionnaires from 366 participants, and all the questionnaires were used in the study. To improve the study’s validity, reliability, and generalizability, a sample size greater than the determined minimum was employed. In addition to increasing statistical power and lowering sampling error, a larger sample guarantees that the various viewpoints of smallholder maize producers are represented. A greater sample size improves the accuracy of SEM results, which leads to more robust hypothesis testing, given the wide variation in climate adaptation behaviours.

#### 2.2.1. Data processing.

The data obtained from questionnaires were processed using Excel and SmartPLS 4 software. The questionnaire data was captured in Excel and exported to the SmartPLS 4 software for further analysis. Structural equation modelling (SEM) was used to analyse data and obtain the TPB results. Each construct (attitude, subjective norms, and perceived behavioural control) was given variables and coefficients based on how its measurement items performed. The different components can be compared to determine how they contributed to irrigation farmers’ intentions by using these coefficients for each construct. The intended outcomes could be utilized to gauge farmers’ willingness to modify their farming methods in response to changing climatic conditions. Finding out how each construct and its assessment items influenced farmers’ decision-making and whether they significantly affected the decisions they made on the farm was imperative. It was also possible to determine the elements that affected the farmers’ decisions and behaviour about water consumption.

### 2.3. Theoretical framework

#### 2.3.1. The theory of planned behaviour.

Developed by Ajzen [24], the Theory of Planned Behaviour framework and constructs are based on earlier decision-making models, namely the Theory of Propositional Control and the Theory of Reasoned Action [25]. It is a socio-psychological model that posits a person’s Intention as the primary predictor of behaviour. According to the Theory of Planned Behaviour, an individual’s intent can be influenced by three factors: attitude, subjective norms, and perceived behavioural control.

**Fig 2:**
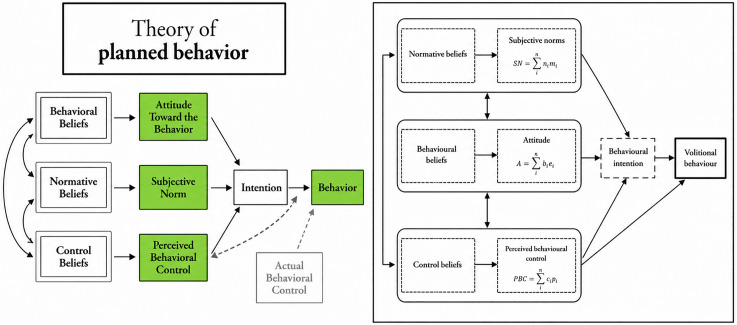
The core components of the theory of planned behaviour.

Fig 2 illustrates the theoretical framework of TPB. Three factors determine Intention. The first is attitude, which is a person’s general assessment of the behaviour. The second is subjective norms, which are a person’s opinions about whether their significant others believe they should act in a certain way. The final metric, known as perceived behavioural control, gauges how much a person believes they have control over their behaviour [26].

One can observe how one construct leads into the next since the three elements affect an individual’s behavioural intent. A person’s behavioural Intention to perform or not determines the actual behaviour they engage in [24]. Numerous research has used the Theory of Planned Behaviour to assess farmers’ attitudes, goals, and adaptive practices [3,5,12,15,27–30]. The TPB was used in this study to evaluate smallholder maize growers’ decisions about irrigation water use, considering climate variability. Structural Equation Modelling is applied in this context to obtain the Theory of planned behaviour-related data, as discussed in the next section.

#### 2.3.2. Structural equation modelling.

The structural equation modelling includes two different variables: observed variables and latent (unobserved) variables. Latent variables are not directly measured but estimated from observed variables, which are directly measured. Eventually, many constructs of Interest are unobservable. In the Theory of Planned Behaviour model briefly described above, behavioural Intention, attitude, subjective norm, and perceived behavioural control may not be directly observed; instead, their latent variables can be calculated from measured indicators. The theoretical framework of the Theory of Planned Behaviour is consistent with the purpose of structural equation modelling for analysis in the sense that structural equation modelling allows a researcher to evaluate an entire model on a “micro level” and to test individual effects [31].

**Fig 3:**
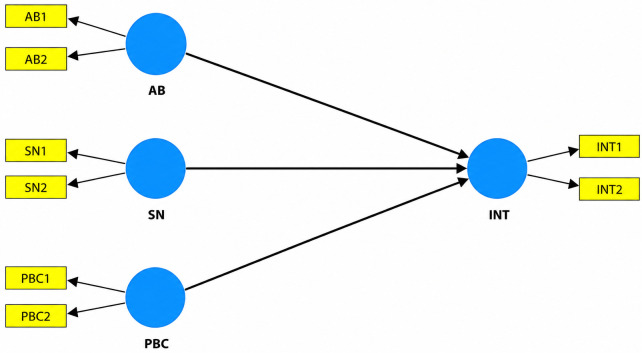
Structural path model diagram exploring irrigators’ Intention for adapting to climate variability.

The SEM of the Theory of Planned Behaviour (TPB) consists of measurement and structural components. The SEM designed for the present study includes the directly observed variables and the unobserved latent variables that are associated with the observed variables. The SEM for the TPB is based on the theoretically grounded causal relationships between the latent variables, such as farmers’ behavioural Intention, attitude, behaviour, subjective norms, and perceived behavioural controls towards exploring irrigators’ Intention for adapting to climate variability. Fig 3 illustrates the hypothesis of the structural part of the model that the farmers’ willingness to adapt to the impacts of climate variability is dependent upon their attitude, behaviour, subjective norms, and perceived behavioural controls.

SmartPLS 4 was used to estimate the path model. The path model and conceptual framework were utilized to test the study’s hypotheses for assessing the variables influencing farmers’ decisions and behaviours in response to climate variability. The hypotheses are as follows:

Hypothesis 1:
  Ho: There is a significant relationship between attitude and the Intention of smallholder maize producers to adapt to climate variability.
  H1: There is no significant relationship between attitude and the Intention of smallholder maize producers to adapt to climate variability.
Hypothesis 2:
  Ho: There is a significant relationship between subjective norms and the Intention of smallholder maize producers to adapt to climate variability.
  H1: There is no significant relationship between subjective norms and the Intention of smallholder maize producers to adapt to climate variability.
Hypothesis 3:
  Ho: There is a significant relationship between perceived behavioural control and the Intention of smallholder maize producers to adapt to climate variability.
  H1: There is no significant relationship between perceived behavioural control and the Intention of smallholder maize producers to adapt to climate variability.

#### 2.3.3. Empirical specifications of the theory of planned behaviour.

This section explores the Theory of Planned Behaviour and the empirical specifications for attitude, subjective norms, perceived behavioural control, Intention, and behavioural Intention in detail. Table 1 presents empirical specifications of TPB. The empirical specifications are based on the Theory of Fishbein and Ajzen [32], which highlights that people’s easily available opinions about an object, a belief being the subjective likelihood that an object possesses a particular quality, determine their assessments of or attitudes toward the phenomenon.

**Table 1:** Empirical specifications of TPB.

Construct	Empirical equation	Description
Attitude	$A=\sum_{\omega }^{a}D_{\omega }G_{\omega }$	Where: A – Attitude Dw– the strength of the belief (subjective probability) that the object possesses the w attribute Gw– the evaluation of attribute w, and the sum is over all attributes. α – number of attributes
Subjective norms	$S=\sum_{w}^{n}B_{w}C_{w}$	Where: S– subjective norm B– normative belief regarding w C– motivation to comply with w
Perceived behavioural control	$PB=\sum_{w}^{a}SwF_{w}$	Where: PB – perceived behavioural control S– the subjective probability or belief that control factor w is present. F– the power of control factor w to facilitate or inhibit the performance of the behaviour
Intention	$INT=\sum_{w}^{r}H_{w}k_{w}$	Where: INT – Intention to perform behaviour. H – the positive or negative Intention to perform behaviour. K – the willingness of control factor w to influence the performance of behaviour
Behavioural Intention	I=IB=(A)ra+(S)rs+(PB)rpb	Where: I - Interest in behaviour IB – behavioural Intention A -attitude to behaviour S – subjective norm to behaviour PB – perceived behavioural control ra – relative weight of A rs – relative weight of S rpb – relative weight of PB

Sources: Authors’ Compilations.

The entire collection of easily available normative ideas about the expectations of significant referents determines the prevailing subjective norm [32]. Perceived social pressure from others to behave in a certain way and the motivation to conform to those people’s opinions determine subjective norms, which are defined as the belief that an influential person or group of people will approve and support a particular behaviour [33].

Perceived behavioural control is influenced by each control factor’s potential to either support or obstruct the performance of an action in direct proportion to the subject’s subjective likelihood of the control factor’s existence [34].

#### 2.3.4. The empirical specifications of structural equation modelling.

This section explores structural equation modelling. This empirical specification, illustrated in Equation 5, indicates maize producers’ behavioural Intention (IBI). It measured the strength of the relationship between behavioural Intention (IBI) and the latent constructs (A, S; PB).


$$\begin{array}{c} IBI=\beta A\times A+\beta S\times S+\beta PB\times PB+\zeta_{\text{1}} \end{array}$$ (5)

Where:

IBI – is an individual’s behavioural Intention.

A – is the latent construct, with β denoting the path coefficient that indicates the strength and direction of the relationship between the latent construct (A) and behavioural Intention (IBI).

S – is the latent construct with β denoting the path coefficient that indicates the strength and direction of the relationship between the latent construct (S) and behavioural Intention (IBI).

PB – is the latent construct with β denoting the path coefficient that indicates the strength and direction of the relationship between the latent construct (PB) and behavioural Intention (IBI).

ζ1 – The error term for Intention (IBI).

## 3. Results

### 3.1. Demographic, farm, and household characteristics of respondents

The demographic, farm, and household profile of respondents in the Tshiombo Irrigation Scheme, as shown in Table 2, highlights several key characteristics. The sample is fairly balanced in terms of gender, with 53% of respondents being female and 47% male. Age distribution reveals that the majority of farmers are older, with 61% aged 51 and above, reflecting an ageing farming population. In terms of education, most respondents (71%) have completed secondary education, while only a small %age (11%) have obtained a diploma or higher education. Farming experience is substantial, with 75% of respondents having more than 10 years of experience, although most have less than 20 years (40%). Interestingly, very few farmers (4%) engage in off-farm work, indicating a strong dependence on farming as their primary livelihood.

**Table 2:** Respondents’ demographic, farm, and household profile.

Variable	Classification	Frequency	Percentage
**Demographic profile**			
Gender	Male	171	47%
	Female	195	53%
Age	21-30	10	3%
	31-40	37	10%
	41-50	94	26%
	51-60	110	30%
	61 and older	115	31%
Highest education level	Primary (Grade 1–7)	65	18%
	Secondary (Grade 8–12)	260	71%
	Diploma	27	7%
	Undergraduate degree	14	4%
**Farming experience**	Less than 10	145	40%
	11-20	127	35%
	21-30	53	14%
	31-40	24	7%
	41-50	16	4%
	51-60	1	0,3%
Off-farm work	Yes	14	4%
	No	352	96%
Farm characteristics profile			
The land ownership type of the farm	Communal	15	4%
	Rented land	4	1%
	Customary land	347	95%
Farm located near modern medical facilities, populated areas, or markets	Yes	58	16%
	No	308	84%
Distance from markets	Less than 1 Km	10	3%
	1-5 Km	55	15%
	6-10 Km	8	2%
	11-15 Km	16	4%
	16-20 Km	60	16%
	More than 20 Km	217	59%
Succession plan	Yes	288	79%
	No	78	21%
Type of farming performed	Crop production	342	93%
	Mixed farming	24	7%
Rural flex irrigation times	Off-peak	63	17%
	Standard	101	28%
	Peak	145	40%
	All	57	16%
Challenges ranked	Water scarcity	1	23%
	Climate change	2	27%
	Lack of access to land	3	29%
	Lack of access to finance	4	24%
	Cost of water	5	27%
	Loadshedding	6	75%
	Electricity	7	78%
Size of farm	Less than 1 Ha	–	–
	1-1.5 Ha	353	96%
	1.51-2 Ha	11	3%
	More than 2 Ha	2	1%
**Household characteristics.**			
Age	21-30	2	1%
	31-40	19	5%
	41-50	52	14%
	51-60	92	25%
	61 and older	201	55%
Male members living in the household	None	9	2%
	One	55	15%
	Two	102	28%
	Three	92	25%
	Four	68	19%
	Five	20	5%
	Six	16	4%
	Seven	4	1%
Female members living in the household	None	6	2%
	One	51	14%
	Two	134	37%
	Three	105	29%
	Four	50	14%
	Five	12	3%
	Six	5	1%
	Seven	2	1%
Male members living in the household are involved in agricultural activities on the farm.	None	36	10%
	One	173	47%
	Two	133	36%
	Three	22	6%
	Four	1	0,3%
	Five	1	0,3%
Female members living in the household are involved in agricultural activities on the farm.	None	32	9%
	One	221	60%
	Two	91	25%
	Three	17	5%
	Four	4	1%
	Five	1	0,3%
Monthly household on-farm income	Less than R 1000	8	2%
	R 1000 – R 5000	72	20%
	R 5000 – R 10 000	69	19%
	R 10 000 – R 15 000	59	16%
	R 15 000 – R 20 000	31	8%
	More than R 20,000	127	35%
Monthly household off-farm income	Less than R 1000	86	23%
	R 1000 – R 5000	190	52%
	R 5000 – R 10 000	48	13%
	R 10 000 – R 15 000	13	4%
	R 15 000 – R 20 000	3	1%
	More than R 20,000	26	7%
Off-farm income	Yes	26	7%
	No	340	93%

Source: Authors’ compilation.

Most farms in the Tshiombo Irrigation Scheme are located on customary land (95%), with only a small proportion being communal (4%) or rented (1%). Most farms are situated far from markets, with 59% of respondents indicating a distance greater than 20 km. Additionally, 79% of respondents reported having a succession plan in place. Crop production is the dominant activity, with 93% of respondents involved, and the most significant challenges ranked by farmers are electricity issues (78%) and loadshedding (75%).

In terms of household composition, 47% of male members participate in agricultural activities, with most households having one or two male members (15% and 28%, respectively). Among female members, 60% engage in agricultural activities, and the largest proportion of female members in households is two (37%). The data also reveals that 35% of households earn more than R 20,000 monthly from on-farm income, while off-farm income remains scarce, with only 7% of households reporting earnings exceeding R 20,000. Notably, 93% of respondents do not engage in off-farm work, indicating a strong reliance on agricultural activities for household income.

Respondents’ Knowledge and Awareness of Climate variability, illustrated in Table 3, shows that a significant majority of respondents possess high or very high knowledge of climate change, with 76% falling into these two categories. Only a small %age reported very low (3%) or low (13%) awareness, while 8% indicated an average understanding. These results suggest that the farmers in the Tshiombo Irrigation Scheme are generally well-informed about climate change.

**Table 3:** Respondents’ knowledge and awareness of climate change.

Classification	Frequency	Percentage
Very low	12	3%
Low	49	13%
Average	28	8%
High	120	33%
Very high	157	43%

Source: Authors’ compilation.

### 3.2. Empirical results

The standard framework of the Theory of Planned Behaviour is concerned with analysing the connections between the constructs pertaining to the adaptation of smallholder farmers in the Vhembe district of Limpopo to climate variability. The outcomes of the initial measurement model assessment, indicator reliability, internal consistency reliability, convergent reliability, and discriminant validity are the five elements of this evaluation. A summary of these assessment results is provided in Appendix A.

#### 3.2.1. Theory of planned behaviour.

**Fig 4:**
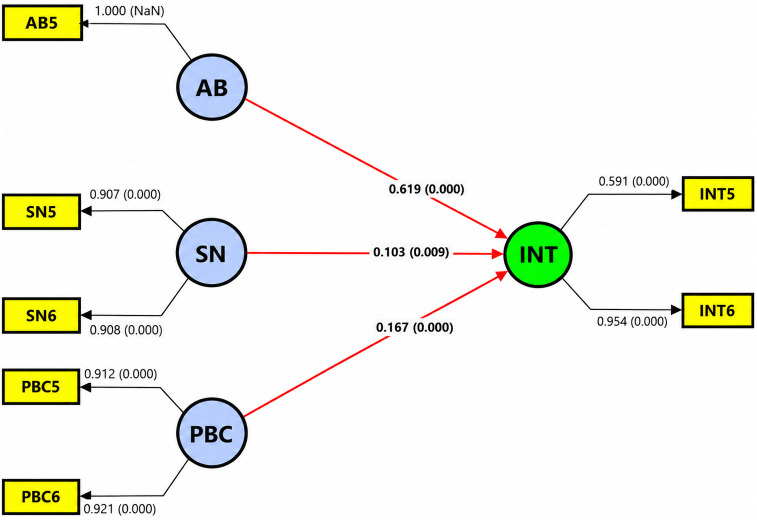
The calculated estimates of the structural model.

Key findings from the evaluation of the measurement and structural models are presented in this section. The model’s standardized estimates are shown in Fig 4. In order to assess the measuring model’s suitability and make sure the constructs appropriately represent the underlying theoretical ideas, the estimations were examined. As explained in the next section, the structural model analysis was carried out following the completion of the measurement model analysis.

#### 3.2.2. Structural model assessment and reporting of the TPB and SEM results.

The next step in the analysis was to evaluate the hypothesised relationships between latent variables by examining the path coefficients (β) and their significance levels (p-values). A bootstrapping procedure with 10,000 resamples was conducted, using the %ile method to determine confidence intervals at a 5% significance level. This approach examined the confidence intervals by evaluating the lower 2.5% and upper 97.5% %iles of the distribution to establish a 95% confidence interval for the coefficients. All hypotheses were evaluated using path coefficients (β), and only those with *p* values≤ 0.05 were considered significant. The hypothesis testing results are shown in Table 4.

**Table 4:** Hypothesis testing results for structural model.

Hypothesis	Relation	Original Sample (O)	Sample mean (M)	Standard deviation (STDEV)	T statistic	P Values	Decision
H1	$A_{B}\to INT$	0.619	0.619	0.032	19.262	0.000	Accept
H2	SN→INT	0.167	0.168	0.043	3.858	0.000	Accept
H3	PBC→INT	0.103	0.105	0.040	2.598	0.009	Accept

Note: *ρ <0.1, ** ρ <0.05, *** ρ <0.01.

Source: Authors’ compilation.

#### 3.2.3. Explanatory power of the model.

The R^2^ value of the standard TPB model indicates the proportion of variance in the dependent variable explained by the independent variables. At the same time, the F-squared statistic measures the effect size of each predictor construct on the outcome. The R^2^ value of 0.466 indicates that approximately 46.6% of the variance in farmers’ intentions to adapt to climate variability is explained by the constructs of attitudes, subjective norms, and perceived behavioural control. This suggests a moderate explanatory power of the model, implying that the selected predictors account for a significant portion of the variation in intentions among small-scale farmers in Tshiombo.

On the other hand, the augmented TPB model illustrated that, for Intentions (INT), the model explains 87.6% of the variance, showing strong predictive power in determining the intentions of small-scale maize irrigators to adapt to climate variability. Attitudes ($A_{B}$) had a lower R^2^ of 6.9%, while Perceived Behavioural Control (PBC) and Subjective Norms (SN) had R^2^ values of 19.1% and 13.5%, respectively, indicating moderate explanatory power for these constructs.

In addition, $f^{\text{2}}$ Values of the standard TPB model further illustrate that the individual effects of each predictor on the dependent variable were values of 0.02, 0.15, and 0.35, representing small, medium, and significant effects, respectively. The $f^{\text{2}}$ value for attitudes ($f^{\text{2}}$ =0.701) indicates a large effect size, suggesting that $A_{B}$ Play a significant role in influencing farmers’ intentions to adapt to climate variability. The $f^{\text{2}}$ value for PBC is 0.051, which is considered a small effect size, suggesting that, while PBC does influence intentions, its impact is relatively minor compared with attitudes. The $f^{\text{2}}$ value for SN is 0.019, which indicates a negligible effect size. The results are then discussed to provide context for the findings.

## 4. Discussion

### 4.1. Demographic, farm, and household characteristics of respondents

Gender is important in adaptation strategies, as this study found that men lead most farming operations. Moreover, this implies that men are the main decision-makers on farms and are more likely to adapt to the impacts of climate variability. However, the significance of gender was demonstrated in the statement by Obi and Maya [35] that women lead female agricultural activity. In contrast, the males move closer to urban areas for better employment opportunities. Contrastingly, Adeboa and Anang [36] found that male-headed farming operations are more successful in adapting new technologies since men are more likely to grow maize, as women typically grow other crops such as vegetables, soybeans, and groundnuts. Moreover, another study by Atube et al. [37] found that females have a lower capacity to diversify their adaptation since they have heavier domestic responsibilities than their male counterparts.

The results revealed that many of the farmers are senior citizens. Also, the increase in age increases the use of traditional adaptation practices. [38] indicate that older farmers are more likely to adapt to the impacts of climate variability and improve sustainable water usage compared to younger farmers, as older farmers are more active in farming activities than the younger generation. Therefore, it is advisable to train young people in these agricultural pursuits in the future. The study’s findings demonstrated how crucial education is to have the necessary information about climate variability. Higher educational attainment had a more positive influence on farmers’ perspectives of climate variability. This supports the Theory that a factor influencing adaptation to climate variation is farmers’ level of education, since they are more likely to employ sophisticated adaptation techniques to enhance their agricultural output and have a greater awareness of climate change [39]. Being aware of climate change increases the probability of uptake of adaptation measures. Farmers who are aware of changes in climatic conditions have higher chances of taking adaptive measures in response to observed changes.

Farming experience is also an important characteristic in that farmers with the most farming experience are most likely to adapt easily to the impacts of climate variability. Since most smallholder farmers are in rural areas, they rely heavily on farming for employment and food security. As a result, farmers’ previous experience is crucial in their appreciation of the costs and benefits of the different technologies, considering their circumstances and resource endowments [40]. This is supported by the study of Adeboa and Anang [36], which established that as one becomes more experienced in farming, the probability of adopting improved farming practices increases. Experienced farmers have a wealth of indigenous knowledge and information about changes in climatic conditions and the best agronomic practices to adopt.

Smallholder farmers rely heavily on agriculture for their employment and means of subsistence; the results demonstrated a high dependence on agriculture for employment. Moreover, smallholder maize production significantly contributes to job creation, rural development, and the eradication of poverty [18].

The results showed that most of the farmers have access to a local market where they can sell their produce for a profit. Moreover, markets give farmers strong incentives to grow cash crops, which can strengthen their resource base and increase their capacity to adapt to climate variability [41]. Consequently, a study by Mkuna and Wale [40] found that distance from markets negatively and significantly affects the use of soil and water conservation adaptation strategies.

In addition, the results revealed that most farmers heavily relied on crop production as their main type of farming. Many smallholder farmers grow food for their consumption, while a minority is focused on the market [42]. Thus, the focus is mainly on crop production, as rural households depend on it for household food consumption. In the Vhembe district, smallholder farmers primarily choose short-furrow (canal) irrigation as their preferred method of irrigation. A schedule often controls water sharing on canal networks. Plot owners in canal systems typically get irrigation water once a week. Water shortages can occasionally occur because of low river flow or infrastructure failures, particularly canals [43]. As a result, the increasing climate variability will increase the need for irrigation as a source of water for crop production because the drier it gets, the higher the demand for irrigation, increasing the fluctuations of flex irrigation times [41].

Equally important, the challenges farmers experience make changing or adapting water conservation techniques on their farms and altering their decision-making difficult. Similarly, a study by Mkuna and Wale [40] confirmed that the increase in adaptation measures is important for smallholder farmers with access to technology such as electricity and machinery. Also, electricity is important, especially for irrigation, as most smallholder farmers get water from dams and rivers that are used for power generation [38]. This shows how vital it is that farmers have a reliable source of electricity to increase their water use adaptation mechanisms.

The importance of irrigation as a successful water adaptation depends on land access and farmer participation; thus, smallholder farmers must have access to land to apply water conservation techniques [43]. This further demonstrates the impact climate variability has on scarce water resources. Thus, by transferring water to its most valuable use while the market places an opportunity cost on that water, demand management techniques like water marketing are required to reduce scarcity and encourage conservation [44].

Smallholder farmers need access to financial assistance to improve their water adaptation to the impacts of climate variability. This is supported by the study done by Atube et al. [37], stating that farmers can invest in more expensive but highly profitable farming practices when they have easier access to financing or cash flows, which may lessen the detrimental impacts of climate change on food production. Consequently, in situations where a large number of people receive free water, significantly worse water shortages arise from uneven distribution, inadequate management, leaks along conveyance structures, disregard for irrigation schedules, and lack of enforcement of water use regulations, resulting in inefficient water use and therefore increases the shortages of water [40].

Smallholder farmers have smaller farm sizes, reducing their willingness to adapt to climate variability, as they have smaller farms that they rely heavily on for sustenance. This is reinforced by the study done by Adeboa and Anang [36], which corroborates that the uncertainty combined with fixed production and information costs means that smallholder farmers are unable to adjust to newly introduced farming technologies beyond a critical farm size. This may be the case because large farms enable the adoption of recently introduced agricultural techniques without requiring a lot of area for traditional farming techniques [37].

The results illustrated that most of the households in the Vhembe district had more than one male family member. Moreover, the significance shown in the monthly household on-farm income confirmed that most smallholder farmers rely heavily on their farming operations for a livelihood and food production. In this regard, [38] found that household income from agriculture is a substantial economic resource for smallholder farmers. Matchaya et al. [44] added that a decline in agricultural production for smallholder farmers would profoundly impact their livelihoods. Thus, the unwillingness of many smallholder farmers to adapt to water-conserving technologies is very high. Smallholder farmers are more equipped to adjust their management methods to climate change and water scarcity because they have access to greater financial and non-financial resources [41].

### 4.2. Structural equation modelling and the theory of planned behaviour

The Theory of Planned Behaviour comprises three constructs: attitude, subjective norms, and perceived behavioural control. Results and hypotheses are discussed for each construct and how they relate to previous similar studies regarding farmers’ intentions to adopt sustainable water use behaviour in response to the impacts of climate variability.

#### 4.2.1. Attitude.

A farmer’s attitude and actions to adapt explicitly influenced by how much they perceive climate variability. Additionally, farmers’ attitudes toward water use affect whether they plan to implement water conservation measures. The findings demonstrated that attitudes have a significant impact on the behaviour of farmers regarding water use. Numerous studies [3,5,6,12,27-30] revealed that attitudes toward a particular behaviour reflect an individual’s overall evaluation of the behaviour and can enhance the purpose of the performer. The findings demonstrated that attitudes have a significant impact on the behaviour of farmers regarding water use. The results are supported by previous studies [6,27,28], which demonstrated that attitude significantly affects the adoption of new sustainable practices.

The results showed that the first hypothesis, according to which attitudes have a major impact on small-scale irrigators’ intentions to adapt to climate change, was validated. This suggests that as the smallholder farmers’ attitudes increase positively, their intentions to adopt water conservation behaviours increase. The path coefficients between attitudes and Intention were significantly positive at β = 0.619, p < 0.01, implying a strong relationship between small-scale farmers’ intentions and attitudes to adapt to climate variability impacts on their maize production. In order to support these findings, Ataei et al. [27] found that farmers’ intentions to implement water conservation measures are influenced by their attitude toward water conservation. This implies that a positive attitude towards water conservation increases the farmers’ Intention to perform and implement water conservation strategies in their maize production.

Moreover, Yazdapanah et al. [5] reported that attitudes are relatively favourable for water conservation adaptation. However, Zamasiya, Nyikahadzoi, and Mukamuri [3] found that although most smallholder farmers believe that the climate is changing, they nevertheless have unfavourable attitudes about recommended climate change adaptation strategies. Therefore, a smallholder farmer’s mindset is crucial in determining how they use water in maize production. Nevertheless, it will ultimately rely on how they perceive behaviours, like climate. To boost farmers’ desire to apply adaptive measures, improving their attitudes toward climate adaptations is necessary to ensure efficient utilisation and distribution of scarce water sources. The following section will discuss the impact of subjective norms on farmers’ Intention to adapt to the effects of climate variability.

#### 4.2.2. Subjective norms.

The findings revealed that farmers experience social pressure to reduce their water use. As a sense of moral obligation and accountability to adopt water use behaviours among farmers, personal norms were also raised. The idea that societal and personal standards reinforce farmers’ intentions and behaviours regarding water use was validated. This means that social and normative impacts on people should be considered while creating interventions for protecting water resources. The second hypothesis was validated, according to which small-scale irrigators’ aspirations to adapt to climate change are greatly influenced by subjective norms. Individual farmers’ intentions can be significantly influenced by how others see adaptation behaviours.

The second hypothesis (H2), which proposed that subjective norms significantly influence small-scale irrigators’ intentions to adapt to climate change, was supported. Subjective norms and Intention had a positive and significant path coefficient (β = 0.103, p < 0.01). This implies that the social setting where smallholder farmers grow maize may influence their participation in a particular adaptation behaviour. A farmer’s decision to adapt to climate unpredictability may be influenced by the societal pressure they encounter. Moreover, subjective norms greatly influence groundwater-saving behaviour [6]. This suggests that the perceptions of others are essential catalysts in adopting climate variability adaptation. Muenratch and Nguyen [6] found that the effects of social pressure and neighbouring farms greatly influence the farmers’ intentions to adopt water conservation practices on their farms.

A study by Yazdapanah et al. [5] also confirmed that farmers feel social pressure to practice water conservation strategies on their farms. Positively, the study by Ataei et al. [27] revealed that policy interventions for the conservation of water resources should target the influence of subjective norms as well as people’s social influences. The following section will discuss the impact of perceived behavioural control on farmers’ Intention to adapt to the effects of climate variability.

#### 4.2.3. Perceived Behavioural Control.

The primary components of perceived behavioural control are the ease of the behaviour, the presence of elements that may facilitate the behaviour, and the perception of control over the behaviour. The feeling of control at the farm is vital for behaviour change; therefore, attempts by policymakers to design and implement any intervention should focus on fostering a sense of empowerment and control. The third hypothesis was validated, in which perceived behavioural control significantly influences smallholder irrigators’ intentions to adapt to climate variability. This implies that farmers are more likely to adopt water-saving behaviours if they feel more in control. The path coefficients between perceived behavioural control and Intention were positively significant at β = 0.167, p < 0.01.

The third hypothesis (H3) was supported, which proposed that perceived behavioural control significantly influences small-scale irrigators’ intentions to adapt to climate change. This implies that when farmers feel they can control their maize yield and water resources, they are more willing to adopt water-saving and climate variability strategies to reduce or mitigate the negative impacts of climate variability on maize production. However, although perceived behavioural control positively correlated with Intention, its significance was less prominent. Consequently, subjective norms and attitudes are relatively more favourable for water conservation. A study by Zeweld et al. [12] confirmed that the perceived usefulness and control of outcomes will significantly influence the intentions of farmers to adopt water conservation practices on their farms. However, the study also mentioned that perceived control of behaviour is also influenced by socio-economic factors such as access to relevant and current climate information.

In addition, smallholder farmers have limited means to adopt new technologies and access financial services, allowing them more control and thus being more willing to implement new climate-conscious practices if they have an alternative support system. As a result, it would help future policymakers to make provisions for reducing the risk aversion of small-scale farmers by increasing the sense of control in sustainable water practices. In this regard, Zamasiya, Nyikahadzoi, and Mukamuri [3] added that farmers felt they could manage water conservation more effectively if they had access to the necessary tools and funding to engage in sustainable water practices.

Contrastingly, Yazdapanah et al. [5] found that the results could not validate the Theory of Planned Behaviour since it emphasises the importance of perceived behavioural control for Intention. Farmers perceive the risk of a water crisis to be high and, therefore, are unwilling to change their perceptions of altering their behavioural practices to be more climate adaptable. Thus, in the pursuit of changing behaviour, policymakers could focus on the perceived risk that smallholders have towards changing traditional practices. Therefore, emphasis should be placed on the benefits of changed water use behaviour to increase positive perceptions regarding sustainable water practices.

## 5. Conclusions

Despite its importance to the South African economy, agriculture continues to be the sector that uses the most freshwater resources when compared to the industrial and domestic sectors. The effectiveness and sustainability of water use in the agriculture sector are thus the subject of numerous discussions. Climate variability is one of the variables that could contribute to South Africa’s growing water constraint. Since they are the main users of water in South Africa, agricultural producers’ water-use behaviour is significantly influenced by climate variability. This makes it imperative to learn more about the decisions farmers make and how climatic variability affects their water-use patterns.

This research advanced knowledge by identifying the factors that influence the water use behaviour of smallholder maize farmers in the Vhembe district, Limpopo, South Africa. Moreover, it assessed the role of smallholder maize production in the pursuit of sustainable water utilisation. While previous research has focused on adaptations to climate change, this study focused on how climate variability impacts water usage and its influence on behaviour, affecting crop productivity and water sustainability. In addition, previous studies have not fully grasped how farmers’ behavioural qualities affect the crops they cultivate and the amount of water they consume in agricultural systems. The study sought to ascertain how climate variability affected smallholder farmers’ water use and how climate variability impacted their decisions to manage water use better.

The study contributed to a better understanding of farmers’ decision-making about water usage and adaptation, and how smallholder farmers’ water use behaviour has changed in response to the effects of climate variability. Irrigation smallholder farmers are expected to be able to modify and enhance their practices to mitigate the impacts of climate variability. Meanwhile, crop intensification and water allocation patterns should also be altered when climate variability peaks. Farmers may also adjust how they use water if they receive better knowledge about the effects of climate change and sustainable agricultural resource management to save limited water supplies. Therefore, farmers’ intentions and behaviours can alter when they understand how climate variability affects water resources and production output. This, in turn, will influence their water use behaviour.

The results show that positive attitudes significantly support the Intention to adopt climate-resilient practices. Social norms also play a key role, indicating that farmers are influenced by the views and behaviours of peers, family, and neighbouring farmers. Perceived behavioural control, shaped by access to resources and confidence in applying water-saving techniques, further strengthens farmers’ Intention to change. However, Intention alone may not lead to behaviour change, especially if structural or knowledge barriers remain.

While the study focused on Intention rather than actual adoption, the findings suggest that targeted support can shift water use behaviour. Efforts to improve knowledge, promote social learning, and offer training opportunities will strengthen farmers’ capacity to adapt. Simply providing infrastructure is not enough. Development efforts must include awareness campaigns, access to agricultural advice, and support systems that help farmers understand and apply sustainable practices. The study was limited to maize producers in one district. Future research could extend to other regions and include other agricultural sectors such as livestock and horticulture. Studies that track long-term adoption will also be valuable for policy and practice. Overall, the research highlights the importance of psychological and social factors in shaping water use behaviour. Understanding how farmers form intentions and respond to climate risks can support more effective strategies for managing water under changing environmental conditions.

The study recommends that it is necessary to improve the way smallholder maize farmers use water. Only when farmers possess adequate knowledge about climate variability can they make well-informed decisions. Also, farmers’ water use behaviour can be altered by improving their perceived behavioural control, which would ultimately motivate their intent to adapt. Better access to information resources must be possible for small-scale farmers to understand water conservation strategies and how they improve maize production. Development practitioners and policymakers should encourage smallholder farmers to adopt sustainable practices by emphasising and reinforcing social capital and agricultural advisory services, as well as using media channels and providing intensive training in capacity building.

Policymakers might concentrate on creating policies that significantly increase smallholder farmers’ access to better technologies, focused training on sustainable water use practices, and financial or technical resources, considering the findings. Giving smallholder maize farmers financial support may encourage them to make the required crop adjustments. Additionally, small-scale farmers can make wise judgments if they have access to reliable and valuable information on the fluctuations in climate conditions. The research identified personal and social norms as two decisive factors that should be considered in intervention strategies. Awareness of demand can be raised by providing information, for example, through large-scale advertising campaigns.

Based on the results, the increase in perceived personal norms may be accompanied by focused signals, such as environmental influence, showing the awareness of need, and these influences may be lessened by using modern irrigation methods, which will demonstrate the outcome efficacy. In order to develop a connection with outcome effectiveness, for example, knowledge of how modern irrigation techniques or appropriate farming patterns can conserve water and energy might improve people’s comprehension of the effectiveness of the outcomes, making it helpful in developing future intervention measures. The effect of Intention on behaviour may be limited, so policy or intervention strategy makers who follow these avenues to increase water conservation intention and behaviour should keep that in mind.

Several limitations should be considered when interpreting the findings. For example, farmers base their arguments on positive self-information, so they may overestimate the benefits of water conservation when relying on their knowledge of these benefits. Therefore, more emphasis should be placed on educating small-scale farmers about the impacts of climate variability and how they can improve and implement water-saving techniques to maximise their maize production while preserving the available water resources. Future research on various geographical settings and cross-cultural research will be helpful.

This study concentrated on behavioural intentions rather than the actual application (adoption) of sustainable behaviours. Although behavioural intentions are a prerequisite for adoption, they are not adequate in and of themselves. Therefore, policymakers and future studies can focus on the actual application of climate adaptation practices. Also, the data of this study only focused on the Vhembe District Municipality, and extensive research in other parts of the country could be exploited.

## Supporting information

S1 Appendix Supplementary file of Appendix A [45]. (DOCX)

S1 File Data set maize behaviours. (XLSX)
