# Supplementary material for: Small-scale irrigators’ intentions to adapt water use behaviour to climate variability in South Africa
Source: PLoS One. 2026 Jul 23;21(7):e0351125. doi: 10.1371/journal.pone.0351125 (PMC13395380; doi:10.1371/journal.pone.0351125)
Supplement: S1 Appendix — (DOCX) [file pone.0351125.s001.docx]

**Appendix A**

***Indicator Reliability***

To ensure the robustness of our measurement model, we followed Hair et al. (2021) guidelines, aiming for outer loadings of 0.7 or higher for strong reliability. Loadings between 0.4 and 0.7 were considered for removal only if their exclusion improved internal consistency and convergent validity. Indicator reliability is crucial as it determines the extent to which the measurement items accurately reflect their respective latent constructs. After the initial estimation of the model, it was found that the factor loadings for all constructs were above the threshold of 0.4, apart from the measurement item ${\boldsymbol{A}_{\boldsymbol{B}}}_{\mathbf{6}}$, which registered a factor loading of 0.364. Given that this loading did not meet the acceptable threshold, the decision was made to drop ${\boldsymbol{A}_{\boldsymbol{B}}}_{\mathbf{6}}$ from the model to enhance the overall reliability and validity of the constructs.

***Internal Consistency Reliability***

To ensure the reliability of the constructs in the model, the internal consistency was evaluated using Cronbach's Alpha and Composite Reliability. According to Hair et al. (2021), values between 0.60 and 0.70 are acceptable for exploratory research, and values between 0.70 and 0.90 are satisfactory for more advanced research. Given the exploratory nature of our study, these slightly lower thresholds were deemed appropriate for evaluating internal consistency. A high level of internal consistency indicates that the items within each construct are closely related and measure the same underlying concept, which is crucial for the validity of the model.

The internal consistency results show that all constructs have Cronbach's Alpha and Composite Reliability values above the minimum threshold of 0.6, except for the Intention construct, where Cronbach's Alpha is 0.485 (Table B2). However, Cronbach's alpha is known to underestimate reliability, particularly when the number of items is small or when indicators have varying outer loadings on the construct (Hair et al., 2021). Given this limitation, Composite Reliability offers a more accurate measure of internal consistency, and all constructs, including Intention, meet the minimum requirement based on their Composite Reliability values.

***Convergent Validity***

Convergent validity was assessed using the AVE to determine the extent to which each construct explains the variance of its indicators. An AVE value above 0.5 indicates that, on average, the construct explains more than half of the variance of its indicators, suggesting adequate convergent validity (Hair et al., 2021). While the Attitudes construct does not have an AVE value due to having only one measurement item, all other constructs demonstrated AVE values greater than 0.5, confirming that their indicators exhibit satisfactory levels of convergent validity (Table B2).

***Discriminant Validity***

Discriminant validity was assessed using both the Fornell-Larcker Criterion and the Heterotrait-Monotrait (HTMT) ratio to ensure that each construct is distinct from the others in the model. According to the Fornell-Larcker Criterion, the square root of the AVE for each construct should be greater than the correlations with any other constructs, indicating adequate discriminant validity. As shown in Table B1, the diagonal values (which represent the square roots of the AVEs) for each construct are greater than the off-diagonal values, confirming that the constructs are sufficiently distinct.

Additionally, the HTMT ratio, which further evaluates discriminant validity, indicates that the values are significantly below the threshold of 0.85 (or 0.9 in more lenient cases) for all pairs of constructs. This is also reflected in Table A1, where the HTMT ratios demonstrate acceptable levels of discriminant validity. Overall, these results indicate that the constructs are adequately distinct from one another.

Table A1: Fornell-Larcker Criterion and HTMT Ratios for Discriminant Validity of the Measurement Model for Small-Scale Maize Irrigators' Adaptation Towards Climate Change in the Tshiombo Irrigation Scheme

| **Fornell-Larcker Criterion** | | | | | **HTMT Ratio** | | | | |
| --- | --- | --- | --- | --- | --- | --- | --- | --- | --- |
|  | **A_B_** | **INT** | **PBC** | **SN** |  | **A_B_** | **INT** | **PBC** | **SN** |
| **A_B_** | 1.000 |  |  |  | $\boldsymbol{A}_{\boldsymbol{B}}$ |  |  |  |  |
| **INT** | 0.651 | 0.793 |  |  | $\boldsymbol{INT}$ | 0.779 |  |  |  |
| **PBC** | 0.141 | 0.267 | 0.916 |  | $\boldsymbol{PBC}$ | 0.157 | 0.487 |  |  |
| **SN** | 0.085 | 0.176 | 0.123 | 0.907 | $\boldsymbol{SN}$ | 0.096 | 0.298 | 0.155 |  |

Note: $\boldsymbol{A}_{\boldsymbol{B}}$- Attitudes, $\boldsymbol{INT}$**-** Intentions, $\boldsymbol{PBC}$-Perceived Behavioural Control, $\boldsymbol{SN}$-Subjective Norms

Source: Authors' compilation

A summary of the final measurement model, including the results of the convergent validity, internal consistency reliability, and discriminant validity assessments, is provided in Table A2.

Table A2: Summary of Results for the Reflective Measurement Model of Small-Scale Maize Irrigators' Adaptation Towards Climate Change in the Tshiombo Irrigation Scheme

| **Latent variable** | **Indicators** | **Convergent validity** | | **Internal consistency reliability** | | **Discriminant Validity** |
| --- | --- | --- | --- | --- | --- | --- |
|  |  | **Loadings** | **AVE** | **Cronbach's Alpha** | **Composite reliability** | **HTMT** |
|  |  | **>0.4** | **>0.5** | **0.6-0.9** | **>0.6** | **Significantly lower than 0.85 (0.9)?** |
| **A_B_** | ${A_{B}}_{5}$ | 1.000 | N/A | N/A | N/A | Yes |
|  | ${A_{B}}_{6}$ | Dropped |  |  |  |  |
| **SN** | ${SN}_{5}$ | 0.907 | 0.824 | 0.786 | 0.903 | Yes |
|  | ${SN}_{6}$ | 0.908 |  |  |  |  |
| **PBC** | ${PBC}_{5}$ | 0.912 | 0.840 | 0.809 | 0.811 | Yes |
|  | ${PBC}_{6}$ | 0.921 |  |  |  |  |
| **INT** | ${INT}_{5}$ | 0.591 | 0.629 | 0.485 | 0.808 | Yes |
|  | ${INT}_{6}$ | 0.954 |  |  |  |  |

Source: Authors' compilation
